# Estimating the Spatial Distribution of Soil Properties Using Environmental Variables at a Catchment Scale in the Loess Hilly Area, China

**DOI:** 10.3390/ijerph16030491

**Published:** 2019-02-10

**Authors:** Chenxia Hu, Alan L Wright, Gang Lian

**Affiliations:** 1 College of Economics and Management, China Jiliang University, Hangzhou 310018, China; 2 Soil and Water Sciences Department, University of Florida-IFAS, Gainesville, FL 32603, USA; alwr@ufl.edu; 3 Zhejiang Environmental Monitoring Center, Hangzhou 310012, China; lian.gang@163.com

**Keywords:** soil properties, spatial distribution, land use, landscape position, Loess Plateau

## Abstract

A comprehensive understanding of the spatial distribution and dynamic changes of soil properties are the basis for sustainable land management. Topography and land use types are key factors affecting soil property variability. This study analyzed the effects of land use types and landscape locations on soil properties, based on data from 111 points of surface soil (0–20 cm) in the Zhujiagou catchment on the Loess Plateau of Northwest China. Soil properties include clay, silt, bulk density (BD), soil organic matter (SOM), total nitrogen (TN) and total phosphorus (TP). Land use types include slope farmland (SFL), terrace farmland (TFL), check-dam farmland (CDL), woodland (WL), shrub land (SL) and grassland (GL). Landscape locations include crest (CT), upper slope (US), middle slope (MS), lower slope (LS) and flat valley (FV). Topographical attributes were divided into primary and secondary (or compound) attributes. Correlation analyses were carried out between soil properties and terrain attribute, and multiple-linear regression models were established to estimate soil properties using land use types and topographic attributes as independents. Results indicated that significant differences in soil properties existed between six land use types, except for bulk density. Higher values of clay, silt, SOM and TN occurred in soils from check-dam farmland, but lower values in soils from shrub land. Significant differences among landscape positions were observed for clay, BD, SOM and TN. Clay, SOM and TN contents on flat valley (FV) positions were higher than those of other positions. Different correlations were found between soil properties and terrain attributes. The regression models explained 13% to 63% of the variability of the measured soil properties, and the model for Clay had the highest R^2^ value, followed by TN, silt, BD, SOM and TP. Validation results of the regression models showed that the model was precise for soil bulk density, but the variation was large and a high smoothing effect existed for predicted values of other soil properties. For TP, the predicted result was poor. Further observations suggested that land use was the dominant factor affecting soil chemical properties. But for soil physical properties, especially for BD, topography was the dominant factor.

## 1. Introduction

As the most important determinants of soil quality, soil properties significantly influence land use and ecological processes [1,2]. Spatial variation of soil properties is a key for dynamic the modeling of ecological and environmental process on the landscape scale. The lack of soil information at a detailed spatial resolution greatly increases the uncertainty of model outputs and becomes an essential limitation for regional land quality appraisals and sustainable land use [3,4,5]. Research on soil property variability across landscapes has been the focal issue of ecological and environmental research since the 1990s [6]. Therefore, characterizing distribution and spatial variability of soil properties based on site characteristics, including climate, land use, landscape position and other variables, is crucial for predicting rates of ecosystem processes [7], understanding evolving mechanisms of ecosystems [8], and further assessing the effects of future land use changes on soil properties [9].

Climate, parent material, topography, and biotic factors influence soil formation [10,11]. However, a large proportion of the local soil variation can be attributed to land use and topography rather than climate and parent material. Land use is regarded as an integrator of several environmental attributes which influence soil properties and change soil structure and nutrient export [12,13]. Land use and soil management practices affect related processes, such as erosion, runoff and leaching [14,15,16,17,18], and consequently, modify the processes of transport and re-distribution of soil particles and nutrients. Significant differences in amounts of runoff, soil loss and nutrient loss were observed between land use and management types [19,20,21,22]. Therefore, land use must be considered when relating soil properties to environmental conditions [13].

Topography is a dominant control of earth surface processes and influences soil chemical and physical properties [23]. Soil development occurs in many landscapes in response to water movement through and over the landscape as subsurface and overland flow. Furthermore, terrain attributes can characterize these flow paths and soil properties. Thus, quantitative information of terrain is widely used in soil studies including the modeling and prediction of soil properties [3,24]. The Loess Plateau of China has the highest rate of erosion in the world, as current surface loss in hilly areas of the Loess Plateau approximates 5000–10,000 Mg km^−2^ year^−1^ [25,26]. Due to severe soil erosion, the ground surface has been incised by rill and gully erosion. Many studies have indicated that topography plays an important role in controlling spatial patterns of soil properties in the loess hilly area [27,28,29,30]. Studying the relationship between soil properties and topographic attributes is of major importance on the Loess Plateau of China because of its distinct variation in topography.

Assessment of spatial pattern is critical in determining soil ecological diversity [30,31] and gauging the effects of anthropogenic activities on soil functions and associated ecosystem services [32,33,34,35]. Recent studies demonstrated the existence of useful predictive relationships between quantitative environmental variables and soil properties [36,37,38,39,40], with the most promising environmental variables being generated by digital terrain analysis and remote sensing. With the development of geographic information science and technology, more relational studies are now conducted at the landscape level. In the Loess Hilly area in China, these kinds of studies are sparse. The aim of this study provided a method of quantitative research for estimating spatial distribution of soil properties, which would help land resource administration to better comprehend spatial variation of soil properties to improve land management practices for regions with data scarcity.

Based on the hypothesis that a wide array of soil properties varies among land use types and landscape positions, and that they are strongly influenced by terrain attributes. The objectives of this study were to: (1) assess the effects of land use and landscape position on soil properties; (2) explore the relationship between soil properties and terrain attributes; (3) identify environmental variables in majorly impacting the spatial distribution of soil properties.

## 2. Materials and Methods

### 2.1. Study Area

**Figure 1:**
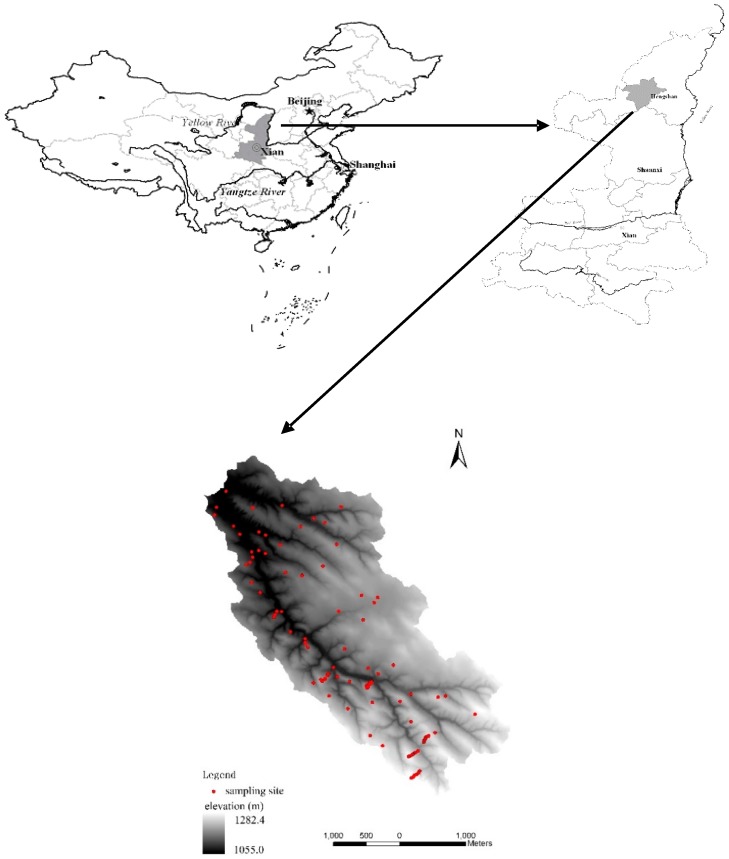
Location of the Zhujiagou catchment and the distribution pattern of sampling sites.

The Zhujiagou catchment (37°56′–37°59′ N, 109°21′–109°24′ E) is located in the Loess Plateau in the northern Shaanxi Province, China (Figure 1). The catchment has an area of 11.3 km^2^, with very steep slopes (more than 50°) and altitudes ranging from 1055 m to 1282 m. There are substantial topographic variations with typical loess hills and gully landforms within the study area.

The region has a semi-arid continental climate with an average annual temperature of 8.6 °C (average maximum 23 °C in July; average minimum −9 °C in January). There are 146 frost-free days and an average of 2815 h of sunshine each year. The average annual precipitation is 403 mm with great interannual variability, as more than 60% of the rainfall occurs between July and September. There is little run-off during the dry season (winter), but high run-off during the rainy season (summer).

Soils of the study area developed on wind-accumulated loess parent material. According to the FAO-UNESCO (Food and Agriculture Organization of the United Nations, United Nations Educational, Scientific and Cultural Organization) soil classification system, the soil is classified as a Calcic Cambisol. The soil in the catchment is characterized by a silt content ranging between 5% and 79% and a clay content ranging between 1% and 12%. The erosion rate is extremely high (10,000–12,000 t km^−2^ year^−1^). Due to serious soil erosion caused by precipitation, the ground surface has been incised strongly with rill and gully erosion.

Land-use categories in the study area mainly are comprised of slope farmland, terrace farmland, check-dam farmland (flat land behind a dam, which has been filled up with silt deposits and normally used for agricultural cropping), orchard, grassland, shrub land and woodland consisting of mosaic patterns. Common cultivated crops are millet (*Panicum miliaceum*), maize (*Zea may* L.), beans (*Phaseolus valgaris*), sorghum (*Sorghum* spp.), potato (*Solanum tuberosum*) and wheat (*Triticum* spp.). Minimal natural vegetation is present due to long-term cultivation. Locust trees (*Robinia pseudoacacia* L.) dominate the woodlands. Grassland is mainly covered by annuals, such as sweet wormwood (*Artemisia annua* L.), annual fleabane (*Erigeron annuus* Pers.) and sandy needle grass (*Stipa glareosa p. Smirn*). Littleleaf peashrub (*Caragana microphylla*) and apple trees (*Malus pumila mill*) grow in the shrub land and orchards, respectively.

### 2.2. Current Land Use Mapping

The QuickBird (pan and multi-spectral image) remote sensing image data of April 2014 was used for generating the land use map of the study area. Before manual interpretation, a pilot field survey was conducted in July 2013 in order to understand the current land uses of the area and to aid interpretation. After the interpretation, a field survey was carried out in July 2014 to validate and update the land use map. The land use map was then prepared for further analysis using ArcGIS 8.3 (Esri, Redlands, CA, USA), and classified into six groups: slope farmland (SFL), terrace farmland (TFL), check-dam farmland (CDL), woodland (WL), shrub land (SL) and grassland (GL).

### 2.3. Soil Sampling and Measurements

Accurate locations of sample sites are crucial for registration of field observations and environmental variables derived from terrain analysis and other sources [37]. Geographic positioning systems (GPS) enabled accurate identification of field samples and correct registration with environmental coverages (e.g., DEM, remote sensing, etc.). Based on the six categories of landscape positions including upper interfluve, lower interfluve, shoulder, upper linear, lower linear, and foot slope used by Brubaker [41], the hillslope was divided into five positions: crest (CT), upper slope (US), middle slope (MS), lower slope (LS) and flat valley (FV). The CT position, relative to the upper interfluve, is the uppermost portion of the hillslope that receives little or no overland flow but may contribute runoff to the down slope position. The US position includes the lower interfluve and shoulder. The MS position and the LS position receive overland flow from the upper slope and contribute runoff to the downslope. The FV represents the base of the hill, water and sediment running off the FV may enter waterways or other water conveyance systems.

The land use map and the topographic map were overlain using GIS 8.3. By combining land use types with characteristics of topography and slope position, 140 sample sites were selected as predetermined sample sites. Due to the limitations of terrain and accessibility, the final number of sample sites were slightly fewer than the predetermined sites. In July 2014, soil samples (0–20 cm) were collected from 111 sites. These sites were distributed throughout the catchment according to different topographic positions and land use (Figure 1) and were located at the predetermined points using high precision GPS (SF-2040G, NavCom) with horizontal accuracy of 0.15 m. At each site, triplicate soil samples were collected and homogenized to be representative of the sampling site. The distance between the triplicate samples was 20 m. Each sampling site was then surveyed, and land use type and spatial characteristics recorded.

The examined soil properties included soil texture (clay and silt), bulk density (BD), soil organic matter (SOM), total nitrogen (TN) and total phosphorus (TP). Standard soil analysis procedures of the Chinese Ecosystem Research Network were used for property determination [42].

### 2.4. Topographic Attributes

Digital elevation models (DEMs) provide basic information for characterizing topographic attributes of a terrain, which are derived from a DEM. A high-resolution DEM with a grid size of 5 m was developed using digital contours, streamlines and spot heights from the nine 1:10,000 topographic map sheets covering the study area.

Topographical attributes can be divided into primary and secondary (or compound) attributes. Primary attributes were directly calculated using the ArcGIS 8.3 program from a DEM based upon terrain analysis. Compound attributes involve combinations of the primary attributes and can be used to characterize the spatial variability of specific process occurring in the landscape [3]. These compound attributes may be derived empirically, or by simplifying equations that describe the underlying physics of the process [3,24].

Primary attributes include elevation (*H*), slope gradient (*β*), aspect (*α*), plan curvature (*K_h_*), profile curvature (*K_v_*); specific catchment area (*Ac*).

Secondary attributes include compound topographic index (*CTI*), stream power index (*SPI*) and sediment transport index (*STI*).

(1) $$CTI=\ln\left(\frac{Ac}{\tan\beta }\right)$$

(2) SPI=ln(Ac×tanβ×100)

(3) $$STI={\left(\frac{Ac}{22.13}\right)}^{0.6}{\left(\frac{\sin\beta }{0.0896}\right)}^{1.3}$$

The secondary indices are parameters related to the surface and subsurface water and sediment transport processes. The compound topographic index has been used to characterize the spatial distribution of zones of surface saturation and soil water content in the landscape [3]. The stream power index is directly proportional to stream power, which is the time rate of energy expenditure and therefore a measure of the erosive power of overland flow. The sediment transport index characterizes erosion and deposition processes and the effects of topography on soil loss. The definition of each terrain parameter and its physical meaning in terms of slope and pedogenic processes are described in Moore [3] and Florinsky [24]. Aspect (degrees clockwise from north), which is a circular variable, was transformed into sin(aspect) and cos(aspect), as recommended by Bourennane [43] and King [44].

### 2.5. Statistical Analysis

A descriptive statistic was used to demonstrate characteristics of soil properties. A one-sample *K*-*S* test was carried out to test the normal distribution of soil properties. A correlation analysis using Pearson correlation coefficients was employed to show correlation between soil properties and terrain attributes. A one-way ANOVA with multiple comparisons (LSD, Least-significant difference) was performed to separately test the influence of land use type and landscape position on soil properties. The relationships between soil properties and land use and terrain attributes were determined using stepwise regression. Land use and terrain attributes were used as independent environmental variables. The classical approach of the “dummy” variables was used for qualitative variables [3,15]. The probability for entry was Pin = 0.05 and the probability for removal was Pout = 0.1. All statistical analyses were conducted using SPSS 23.0 (IBM, Armonk, NY, USA).

In order to evaluate the performance of the regression models for different soil properties, all data were divided into two sets, one for regression and another for validation. 20 samples were randomly selected as a validation set, while the other 91 samples were used to build the regression model.

The effectiveness of the regression model was assessed by computing indices from the measured and predicted values. The two indices used were the mean prediction error (MPE) and the root mean square prediction error (RMSPE) [45]:

(4) $$MPE=\frac{1}{n}\overset{n}{\underset{j=1}{\sum }}\left(z_{j}-{\hat{z}}_{j}\right)$$

(5) $$RMSPE=\sqrt{\frac{1}{n}\overset{n}{\underset{j=1}{\sum }}{\left(z_{j}-{\hat{z}}_{j}\right)}^{2}}$$

The MPE measures the bias of the model prediction and an MPE of zero is indicative of an unbiased method. The MPE indicates whether the model is producing estimates that are overestimating or underestimating the observed values. The RMSPE measures the average precision of the prediction.

## 3. Results and Discussion

### 3.1. Descriptive Statistics of Soil Properties

Descriptive statistics of the soil properties are shown in Table 1. Compared with other analogous sites on the Loess Plateau, clay content was low in the study area, and mean silt content was 40%. Desertification is serious in the area due to the proximity of the Maowusu desert. Soil nutrients, including soil organic matter, total nitrogen and total phosphorus, exhibited low concentrations. Coefficients of variation for soil properties were moderate, and all variables met the conditions for normal distribution (One-sample *K*-*S* test).

Many measurements in this study were significantly intercorrelated (Table 2). Clay, silt, SOM and TN were positively correlated, but they were negatively correlated with BD. No significant correlation between TP and other soil properties was found. Higher correlation of TN and SOM may be explained by the SOM contribution to the total nitrogen pool.

### 3.2. Effects of Land Use on Soil Properties

There were statistically significant differences in soil properties between the six land use types, except for bulk density (Table 3). The mean clay content varied between 1.2% and 9.6%. Multiple comparisons revealed that the clay level under check-dam farmland was significantly higher than other land use types. However, the clay level under shrub land was significantly lower compared with other land use types. Contents of silt (15.7–67.8%) displayed similar patterns as clay in the multiple comparisons. High clay and silt contents in check-dam farmland may result from the deposition of sediments carried by overland flow. The high sand content of the shrub land can be explained due to its location on the top of the hill slopes where clay and silt are lost easily due to soil erosion. Furthermore, the high sand content may result from the influence of desertification to a certain degree. The spatial variation of erosion intensity was generally controlled by topography and increased from the top to the bottom of the slopes [46]. There was no statistical difference in bulk density among different land use types. The coefficient of variation and spatial variation of bulk density was low in the study area, which may result in small differences in the average values of BD among land use types.

The mean SOM content varied between 1.55 and 7.53 g kg^−1^. Multiple comparisons revealed that SOM levels under check-dam farmland was significantly higher than under other land use types, and the SOM content under shrub land was the lowest among all land use types. However, compared with other areas of Loess Plateau studied by Zheng [47], the mean SOM content (4.47 g kg^−1^) was lower in this catchment. This may result from a combination of serious soil erosion and low C inputs. In our prior study [48], crop residues like millet and soybean stalks were fed to animals, while maize and sunflower stalks were used as fuel for cooking. A little amount of residue was returned to the cropland, resulting in low C inputs. Although TN exhibited a small coefficient of variation, it was significantly different among the six land use types. The TN content displayed the similar patterns as SOM in the multiple comparisons, and this similarity may be associated with SOM influencing nutrient retention and supply [41].

The TP contents ranged from 0.61 to 1.30 g kg^−1^. No significant difference in TP content was observed between terrace farmland and check-dam farmland, but the TP content of terrace farmland was significantly higher than elsewhere. The most important reason for higher TP in terrace farmland may be low runoff potential, which decreases loss of P in terrace farmland. On the other hand, this result can be attributed to better farming conditions (the local farmers prefer to use more fertilizer on terrace farmland). There was no significant difference in TP between the other of land use types. Most of P is held very firmly in crystal lattices in largely insoluble forms, such as various Ca, Fe and AlPO_4_, and is also chemically bonded to the surface of clay minerals [49], which may result in relatively small differences in the average values of TP among land use types.

For most of the soil properties, there were no significant differences between slope farmland, woodland and grassland. This result was not consistent with other studies [50,51], which found that woodland and grassland had higher SOM and TN than slope farmland. This may be the result of the change in land use policies since 1999. In order to return to the natural regulating functions of land resources, the Chinese government launched the Green-for-Grain program, aimed at returning barren slope farmland to woodland and grassland. In the study area, however, converting woodlands and grasslands from slope farmland is unable to effectively control soil and water loss because of the low survival ratio of the trees and the short growth period. Furthermore, the former woodlands are sparse, and few plant residues persist.

### 3.3. Soil Properties Associated with Landscape Position

Significant differences among landscape positions were observed for clay, BD, SOM and TN (Table 4). Results indicated that clay, mean SOM and TN contents were higher on FV position than for other positions. The lowest clay content was for the CT position, and multiple comparisons of clay revealed that the clay content on the FV position was significantly higher than for the CT and LS position. This result may be attributed to surface run-off and soil erosion. On the one hand, the CT position is the uppermost position of the hillslope and a large amount of clay can be transported to downslope positions by surface run-off with the result that soil structure at the CT position becomes more compact. Therefore, the highest clay content appeared at the FV position. The highest BD occurred at the CT position and was significantly higher than other landscape positions except for the FV position. Similar results were detected by Malo [52] and in general, the high soil bulk density is a signal of land degradation [53].

Soil erosion is an important process influencing nutrient loss from ecosystems [19], and surface runoff is the major transport mechanism for soil nutrient losses in the Loess Hilly area [54]. Soil organic matter content varied with position on the slope [55], as the lowest SOM and TN occur at the LS position, which was significantly lower than SOM content at the FV position. The highest value of SOM and TN occurred at the FV position. Although differences for silt and TP among landscape positions were not statistically significant, further observation suggested that they tended to have higher value at the FV position and lower value at the CT position, with similar patterns existing for clay, SOM and TN. The higher values of soil properties occurring at the FV position may result from deposition of soil organic matter, and nutrients eroded from upslope positions.

### 3.4. Relationship between Soil Properties and Terrain Attributes and Key Environmental Variables in Impacting Spatial Distribution of Soil Properties

The correlation analysis (Table 5) showed that clay and silt had positive correlations with *β* and *K_h_*, and negative correlations with *CTI*. *K_h_* is a measure of convergence of substance flows, and soil moisture and lateral intrasoil flow increase if *K_h_* < 0 and decrease if *K_h_* > 0 [24,56]. This result led to positive correlations of clay and silt with *K_h_*. A negative correlation was found between BD and *β*, but BD had a positive correlation with *CTI*. SOM had a negative correlation with *CTI*, *SPI* and *STI* and TN had a negative correlation with *STI*. The secondary indices, *CTI*, *SPI* and *STI* are parameters related to surface and subsurface water and sediment transport processes. They reflect the spatial distribution of zones of surface saturation and soil water content in landscape and describe potential flow erosion and related landscape processes. All these comparisons resulted in positive or negative correlations between the secondary indices and some soil properties. There was no significant correlation between TP and most topographic attributes, except a negative correlation with *β*. Slope gradients influence infiltration, drainage and runoff, and steeper slopes may exhibit lower soil moisture owing to lower infiltration rates, rapid subsurface drainage and higher surface runoff [57]. These factors influenced redistribution of soil properties, especially for TP, because soil phosphorus loss is mainly induced by runoff.

The correlations between terrain attributes and soil properties indicated that the soil developed in response to the way water flowed through and over the landscape. On the whole, the correlations between terrain attributes and soil properties were relatively weak, and this result was unexpected. Surface soil properties were mostly modified by land management and as McKenzie and Austin [36] noted, relationships between landform and soils are more strongly expressed in younger alluvial units than in older landscape units.

To explore the influence of environmental variables on soil properties, land use types and topographic attributes were used to explain the variation of measured soil properties using multiple stepwise linear regression. The regression equations presented in Table 6 explain 13% to 63% of the variability of measured soil properties (Table 6). Regression models for clay had the highest R^2^ value, followed by TN, silt, BD, SOM and TP. With higher resolution, larger scale digital elevation models and more detailed environmental variables, it may be possible to explain a higher proportion of the variance. However, because of high spatial variability of soil properties and unique physical conditions of the loess plateau, it is unrealistic to expect that the methods employed could explain more variance.

Further observations suggested that land use was the dominant factor affecting soil chemical properties. For example, land use and topographic attributes explained 24.7% and 9.7% of the variability for SOM, respectively. For TN, land use and topographic attributes explained 45.6% and 5.6% of the variability, respectively. For soil physical properties, topography was a dominant predictive factor, especially for BD. Land use and topographic attributes explained 23.2% and 39.5% of the variability for clay, respectively. For silt, land use and topographic attributes explained 29.8% and 15.2% of the variability, respectively. These results imply that change of soil physical properties is a long-term process, but soil chemical properties can be changed by human activities in the short term.

**Figure 2:**
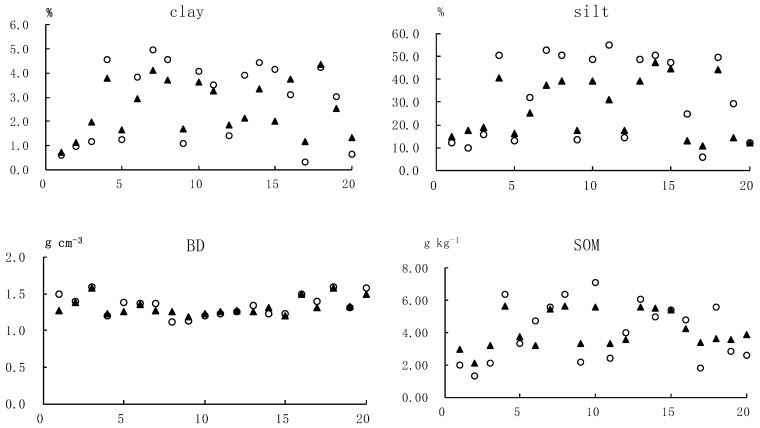

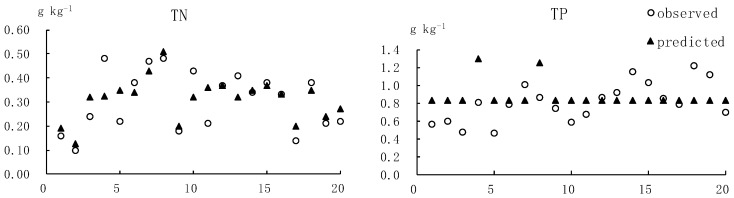
Comparison of observed and predicted value of soil properties.

The mean prediction error (MPE) and the root mean square prediction error (RMSPE) measuring the bias or precision of prediction models should be as small as possible for unbiased and precise prediction. For all soil properties except TP, MPE and RMSPE were relatively small. A comparison of observed and predicted values of the linear regression model showed that the regression model was precise for soil bulk density (Figure 2) with the MPE and RMSPE being 0.009 and 0.127, respectively. For other soil properties, there was a high smoothing effect on the predicted values, and the variation between the predicted and observed values was larger compared to soil bulk density. This observation may be attributed to less spatial variability of bulk density. For TP, the predicted result was very poor and only TEL entered the regression model which explained 12.8% of the variability. This result indicates that some other stochastic factors such as human activity may affect TP to some extent, and therefore needs further study.

## 4. Conclusions

This study showed that land use types and landscape positions had variable effects on soil properties. Soils under six land use types exhibited significant differences in several properties, except for bulk density. Higher values in clay, silt, SOM and TN were measured in soils from check-dam farmland and lower values were observed in soils from shrub land. The TP content of terrace farmland was significantly higher than other land uses. Significant differences among landscape positions occurred for clay, BD, SOM and TN. The highest values for clay, SOM and TN occurred at FV position located the bottom of the slope.

Different correlations were found between soil properties and terrain attributes. Clay and the silt had positive correlations with *β* and *K_h_*, and negative correlations with *CTI*. The BD had a negative correlation with *β*, and a positive correlation with *CTI*. The SOM had negative correlation with *CTI*, *SPI* and *STI* and TN had a negative correlation with *STI*. However, no significant correlation was found between TP and most topographic attributes. Correlations between terrain attributes and soil properties indicated that soil develops in response to the way water flows through and over the landscape. Topography and land use types were primary factors affecting soil property variability for catchment scale in the loess hilly area. Land use type was a dominant variable affecting soil chemical properties but topography was a dominant factor affecting soil physical properties. Slope and *CTI* were efficient variables for estimating soil physical properties.

The availability of high-resolution digital elevation models and remote sensing can provide an efficient, quantitative method for modeling and forecasting spatial variability of soil properties at smaller scales. Soil is a complex system with obvious temporal and spatial characteristics. Landscape positions impact runoff, drainage, soil temperature, and soil erosion and consequently soil formation. Along a hillslope, differences in soil formation caused differences in soil properties. This study was a methodological attempt for estimating soil properties using regression models. But interrelationships between environmental variables were not considered for this study. Further research is needed to more comprehensively understand the interactive relationships among landscape position, soil erosion, soil properties, and land management.

## Figures and Tables

**Table 1:** Descriptive statistics for soil properties.

Soil Properties ^1^	Clay (%)	Silt (%)	BD (g cm^−3^)	SOM (g kg^−1^)	TN (g kg^−1^)	TP (g kg^−1^)
Mean	3.50	40.16	1.26	4.47	0.36	0.85
Median	3.96	47.47	1.27	4.63	0.36	0.78
Minimum	0.25	3.79	1.12	1.30	0.11	0.37
Maximum	10.70	76.26	1.42	9.24	0.87	1.50
Range	10.46	72.48	0.31	7.94	0.76	1.13
Standard deviation	1.71	16.34	0.08	1.69	−0.13	0.26
Coefficient of variation (%)	48.90	40.69	6.33	37.86	37.26	30
One-Sample *K*-*S* Test	1.49	1.86	0.62	0.66	0.49	1.24

^1^ BD-bulk density; SOM-soil organic matter; TN-total nitrogen; TP-total phosphorus.

**Table 2:** Pearson correlation coefficients between soil properties.

Soil Properties ^1^	clay	silt	BD	SOM	TN	TP
clay						
silt	0.92 **					
BD	−0.60 **	−0.70 **				
SOM	0.58 **	0.58 **	−0.31 **			
TN	0.75 **	0.63 **	−0.38 **	0.86 **		
TP	0.14	0.17	−0.19	0.20	0.09	

** Correlation is significant at the 0.01 level (2-tailed). ^1^ See Table 1 for abbreviations.

**Table 3:** Comparison of soil properties for different land use types.

Land Use ^2^	Soil Properties ^1^					
	Clay (%)	Silt (%)	BD (g cm^−3^)	SOM (g kg^−1^)	TN (g kg^−1^)	TP (g kg^−1^)
SFL	3.32 ac	39.50 ac	1.23 a	4.17 ab	0.33 a	0.77 a
TFL	3.24 bc	39.47 bc	1.29 a	4.97 abc	0.35 ab	1.30 b
CDL	9.56 d	67.81 d	1.27 a	7.53 c	0.81 c	0.88 ab
WL	2.46 ab	30.01 ab	1.26 a	3.77 b	0.31 a	0.88 a
SL	1.22 b	15.72 b	1.30 a	1.55 d	0.13 d	0.61 a
GL	3.79 c	43.97 c	1.29 a	4.88 a	0.39 b	0.87 a
*F* value	12.61 ***	4.66 ***	2.22	5.23 ***	12.10 ***	3.37 **

Values in each column with the same letter (a, b, c, d) are not significantly (*p* < 0.05, LSD) different among land use. *, **, ***, Significant at the 0.05, 0.01 and 0.001 level, respectively. ^1^ See Table 1 for abbreviations. ^2^ SFL—slope farmland; TFL—terrace farmland; CDL—check-dam farmland; WL—woodland; SL—shrub land; GL—grassland.

**Table 4:** Comparison of soil properties at different landscape positions.

Landscape Position ^3^	Soil Properties ^1^					
	clay (%)	silt (%)	BD (g cm^−3^)	SOM (g kg^−1^)	TN (g kg^−1^)	TP (g kg^−1^)
CT	3.00 a	34.64	1.34 a	4.59 ab	0.36 ab	0.08
US	3.70 ab	43.76	1.24 b	4.57 ab	0.37 ab	0.90
MS	3.85 ab	44.15	1.22 b	4.72 ab	0.38 ab	0.81
LS	3.14 a	37.93	1.25 b	3.82 a	0.31 a	−0.85
FV	5.31 b	46.19	1.27 ab	5.64 b	0.49 b	0.88
*F* value	1.89 *	0.93	1.42 *	1.31 *	1.82 *	2.86

Values in each column with the same letter are not significantly (*p* < 0.05, LSD) different among landscape position. * Significant at the 0.05 level. ^1^ See Table 1 for abbreviations. ^3^ CT—crest; US—upper slope; MS—middle slope; LS—lower slope; FV—flat valley.

**Table 5:** Correlation matrix of soil properties and terrain indices.

Soil Properties ^1^	*H*	*β*	cos*α*	sin*α*	*K_v_*	*K_h_*	*CTI*	*SPI*	*STI*
clay	−0.17	0.28 *	0.24 *	−0.01	−0.11	0.27 *	−0.42 **	−0.09	−0.15
silt	−0.07	0.32 **	0.21	−0.09	−0.07	0.32 **	−0.52 **	−0.15	−0.20
BD	0.13	−0.34 **	−0.12	0.10	−0.01	−0.10	0.43 **	−0.09	−0.02
SOM	0.03	−0.10	0.07	0.04	−0.12	0.12	−0.23 *	−0.31 **	−0.29 **
TN	−0.10	−0.02	0.20	0.11	−0.09	0.11	−0.22	−0.21	−0.23 *
TP	0.09	−0.17 *	−0.10	0.06	0.12	−0.12	0.06	0.15	0.05

* Correlation is significant at the 0.05 level (2-tailed). ** Correlation is significant at the 0.01 level (2-tailed). ^1^ See Table 1 for abbreviations.

**Table 6:** Stepwise multiple regression function for each soil property.

Dependent Variables	clay	silt	BD ^1^	SOM	TN	TP
Constant	5.87	74.65	1.15	0.71	0.05	830.70
Independent variables						
*H*						
*β*						
cos*α*	0.41 (5) ^4^					
sin*α*	−0.59 (4)	−4.81 (4)				
*K_v_*			−0.01 (2)			
*K_h_*						
*CTI*	−0.49 (2)	−6.39 (1)	0.02 (1)			
*SPI*				−0.04 (3)	−0.002 (3)	
*STI*						
SFL ^2^						
TFL						469.44 (1)
CDL	7.10 (1)	37.99 (2)		0.39 (2)	0.05 (1)	
WL						
SL				−0.22 (1)	−0.02 (2)	
GL	0.92 (3)	9.26 (3)		0.08 (4)	0.006 (4)	
Model						
Sum of squares	139.94	9137.78	0.11	0.75	0.007	635,358.1
Df	5	4	2	4	4	1
Mean square	27.99	2284.45	0.06	0.19	0.002	635,358.1
*F*-ratio	23.82	14.74	14.03	9.42	18.91	11.05
*p*-Value	0.000	0.000	0.000	0.000	0.000	0.001
Residual						
Sum of squares	83.42	11,156.65	0.17	1.43	0.007	4,314,257
Df	85	86	88	86	86	89
Mean square	1.18	154.95	0.004	0.02	2	57,523.42
*R* ^2^	0.63	0.45	0.40	0.34	0.51	0.13
Standard error	1.08	12.45	0.06	0.14	0.009	239.84
Validation						
*MPE*	−0.15	0.58	0.009	−0.04	−0.008	−343.18
*RMSPE*	1.97	2.32	0.13	0.17	0.02	354.54

^1^ See Table 1 for abbreviations. ^2^ See Table 3 for abbreviations. ^4^ Number in parentheses indicate the order in which the variables were brought in to the regressions.
